# Reconstructing the Trophic History of an Alpine Lake (High Tatra Mts.) Using Subfossil Diatoms: Disentangling the Effects of Climate and Human Influence

**DOI:** 10.1007/s11270-018-3940-9

**Published:** 2018-08-15

**Authors:** Lucia Sochuliaková, Elwira Sienkiewicz, Ladislav Hamerlík, Marek Svitok, Dana Fidlerová, Peter Bitušík

**Affiliations:** 1 Department of Biology and Ecology, Faculty of Natural Sciences, Matej Bel University, Tajovského 40, 97401 Banská Bystrica, Slovakia; 2 Institute of Geological Sciences, Polish Academy of Sciences, Twarda 51/55, 00-818 Warszawa, Poland; 3 Department of Biology and General Ecology, Faculty of Ecology and Environmental Sciences, Technical University in Zvolen, T. G. Masaryka 24, 960 53 Zvolen, Slovakia; 4 Department of Ecosystem Biology, Faculty of Science, University of South Bohemia, Branišovská 1760, 370 05 České Budějovice, Czech Republic; 5 Water Research Institute Bratislava, L. Svobodu 5, 812 49 Bratislava, Slovakia

**Keywords:** Bacillariophyceae, Quantitative reconstruction, Total phosphorus, Tourist impact, climatic change, fish population

## Abstract

Diatom analysis was undertaken on a 200-year sediment record in an alpine lake (Popradské pleso, Tatra Mountains, Central Europe). Due to its remote character and well-documented human influence since the mid-nineteenth century, it allows a study of the relationship between anthropogenic pressures and diatom assemblages. Altogether, 122 diatom taxa of 40 genera were identified, and two major taxonomic shifts were revealed in the stratigraphic record. The timing of the first significant shift in ~ 1850 precludes the possibility of being caused by direct human activities, since according to historic documents there was neither continuous human presence nor grazing in the valley before that time. In addition, the direct effect of organic pollution early in the 1960s connected with the operation of a tourist hotel was not clearly reflected in the diatom signal. The diatom-inferred total phosphorus (DI-TP) reconstruction indicated the highest TP content well before the most direct wastewater pollution from a newly built hotel. There was a considerable effect of climate to diatom assemblage structure as well as diatom life forms. Our results suggest that direct organic pollution influenced the diatom communities less than expected, and the main driver of change was climate warming. We hypothesize that it is because of the short residence time of the lake, since it has both strong inlet and outlet, and it has been showed that the inlet had significant effect on benthic communities in the past. At the same time, fish manipulation could have been the reason for some fluctuation in DI-TP unrelated to climate and organic pollution.

**Electronic supplementary material:**

The online version of this article (10.1007/s11270-018-3940-9) contains supplementary material, which is available to authorized users.

## Introduction

Alpine lakes, despite their geographical isolation and inaccessibility, are increasingly affected by changing environmental conditions and human-induced issues. Recently, cultural eutrophication has been representing a major human impact on lakes at global scale (Smol 2002), but its effects interact with the increasingly warming climate that changes nutrient dynamics and, in turn, aquatic biota, especially in mountainous areas (e.g., Catalan et al. 2002; Battarbee et al. 2012 and references therein). The expected effects of climate warming on alpine lakes include changes in lake-water temperature, ice cover duration, retention time, light regime, and water level, as well as changes in the productivity and structure of lake ecosystems (Korhola et al. 2002). Thus, by altering boundary conditions, climate change may shift the baseline of previously defined reference conditions (Battarbee et al. 2005; Thies et al. 2012) and requires setting up new conditions for lake restoration purposes.

Many mountain areas are currently under some type of protection to maintain their unique natural environment with relatively undisturbed ecological conditions. Lakes located in the Tatra Mountains, Central Europe, are part of the Tatra National Park, and for 60 years, fishing, boating, and sheep and cattle grazing in the lake catchments have been prohibited. Despite these restrictions, we are still witnessing the deterioration of water quality of some lakes in the national park, caused mainly by anthropogenic impacts. Lake Popradské pleso, situated in the Slovak part of the Tatra Mts., is a good example of such lake. The area was not populated for a very long period and was only visited by hunters, miners, herbalists, and prospectors. In addition, the valley of Popradské pleso is very steep and rocky, alpine meadows are rare, and thus it is not appropriate for grazing. Since the end of the nineteenth century, when the first small tourist hut was built at the lake’s shore, the tourist infrastructure in the surroundings expanded resulting in a gradually increasing number of visitors, and consequently, higher anthropic pressure (Bohuš 2005). For many years, the infrastructure was built without wastewater treatment systems, and most of the organic waste ended in the lake or its vicinity. Moreover, there are indications that the lake with a natural population of brown trout was subject to repeated and uncontrolled fish manipulations. It has been shown that introduction of fish to oligotrophic lakes can influence the lake biota through nutrient dynamics (Vanni 2002), but also indirectly, via differential predation pressure on zooplankton that alters the phytoplankton community structure (e.g., Jeppesen et al. 2003; Sarnelle and Knapp 2005). Thus, it is reasonable to suppose that the increasing human influence enhanced the trophic status of the lake and altered the structure of aquatic communities. Paleolimnological techniques offer a way to study the lake response to historical environmental changes and impacts of human activities (Smol 2002). Using lake sediment from Popradské pleso, Hamerlík et al. (2016) analyzed subfossil chironomids to reveal their response to eutrophication induced by human activities and climatic oscillations. It is likely, therefore, that other components of the ecosystem, e.g., the plankton, responded similarly or even more strongly.

Thus, we analyzed subfossil diatom assemblages from the sediment record of lake Popradské pleso to track the lake development along a combined gradient of organic pollution, fish manipulation, and climate change over the last 200 years. Diatoms are key indicators of trophic status of lakes and are frequently used in paleolimnological studies to infer environmental variables (Hall and Smol 2010). We hypothesized that the diatom-inferred TP levels would closely reflect the human-induced events both in the lake and its catchment.

## Material and Methods

### Study Site

**Fig. 1:**
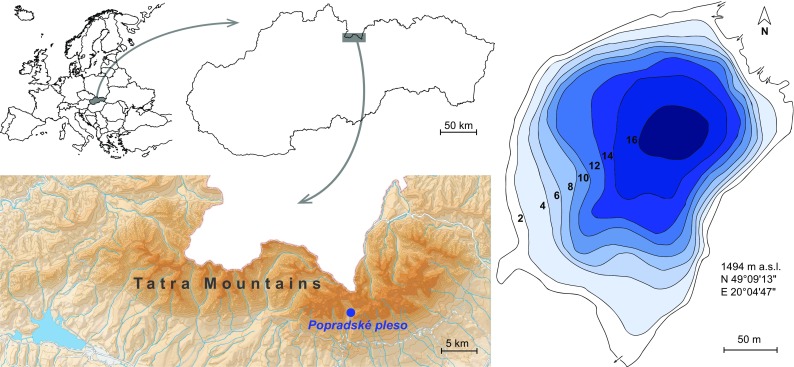
Location of the study lake, Popradské pleso, within Europe, Slovakia and the High Tatra Mts., and the bathymetry of the lake

Popradské pleso (N 49°09′13″, E 20°04′47″) is a subalpine, dimictic lake located just on the tree line at 1494 m a.s.l. (Fig. 1). It has an area of 6.9 ha and a maximum depth of 17.6 m. The catchment of the lake is situated mostly in the alpine zone and consists mainly of bedrock, debris, and alpine meadows. Only a small part of the catchment around the lake is covered by coniferous forest (*Picea abies*) and dwarf pine (*Pinus mugo*). Popradské pleso is the only lake on the Slovak side of the Tatra Mts. with a native population of brown trout (*Salmo trutta*).

**Table 1:** Summary binomial GLMs testing the effect of temperature, DI-TP, and their interaction on the proportions of diatom life forms. Test statistics (F_df,resid. df_) and probabilities (p) are displayed. Significant results are in bold

	Benthic diatoms		Planktonic diatoms		Tychoplanktonic diatoms	
Source of variation	F_1,13_	*p*	F_1,13_	*p*	F_1,13_	*p*
Temperature	**6.81**	**0.0216**	**7.26**	**0.0184**	**6.69**	**0.0226**
DI-TP	2.01	0.1793	0.45	0.5142	2.58	0.1321
Temperature × DI-TP	0.26	0.6190	2.65	0.1276	0.05	0.8211

Water chemistry is characterized by a well-developed carbonate buffering system with high acid neutralizing capacity (> 100 μmol.L^−1^) tightly related to a high concentration of Ca^2+^ and Mg^2+^and pH 6.7 on average. The lake was resistant to acidification occurring in the Tatra Mts. lake district in the second half of the twentieth century. Concerning nutrients, the concentrations of total phosphorus (TP) and total nitrogen (TN) in the Tatra lakes are generally correlated with the amount of soil pool and vegetation density in the catchment (Kopáček et al. 2000). As rocks cover the majority of the catchment of Popradské pleso, nutrient concentrations resemble of that in lakes situated at higher elevations. However, TP concentration is usually higher in Popradské pleso than that in similar Tatra lakes which is usually attributed to human influence (see Bitušík et al. 2006 for more details).

Human activities during the modern history of the lake are well documented (~ 200 years; Juriš et al. 1965; Bohuš 2005). Almost no human actions were observed in the lake’s catchment before the mid-nineteenth century, when tourism appeared in the valley and the lake gradually became an important hub of tourist routes. The development of infrastructure started in 1879 with the building of the first cottage on the lake shore. Since then, new cottages were built, burned/torn down, and rebuilt. All the cottages built in this period were small and had no wastewater systems. However, there is evidence that sewage drained to the lake from the huts (Balon 1964), and that kitchen waste was discharged to the lake (Dyk 1960). The main organic pollution began in 1958 when a big hotel able to host about 150 visitors started to operate close to the lake. Similar to the previous infrastructure, it was constructed without a sewage treatment plant, and the hotel laundry increased the quantity of detergents directly discharged into the lake. A sewage system came into operation in 1962, but its cleaning performance was poor and during winters, due to harsh temperature conditions, it was usually out of order. Moreover, the wastewater pipeline was still discharged into the lake close to the hotel. In 1994, the pipeline was transferred further from the hotel close to the outlet. In 2011, another cottage situated close to the hotel was put into operation.

### Coring and Dating

Two short sediment cores (34.5 cm) were taken from the lake on May 31, 2013 using a Kajak gravity corer from the deepest point of the lake (12 m). The cores were subsampled at 0.5 cm intervals on site, stored in plastic zip-bags, and kept in a refrigerator at 4 °C for later analysis. Sediment samples were dated using the ^210^Pb method and confirmed by the ^137^Cs method. A depth-age model of the 0–8 cm section of the sediment based on ^210^Pb and ^137^Cs dating is presented in the Supplementary Material (Fig. S1). Sedimentation rates for each sample were calculated using the method described in Appleby (2001). For more details on the dating and sediment properties, e.g., organic matter content (% LOI) and element concentrations, see Hamerlík et al. (2016).

### Temperature Data

Historical air temperatures (1814–1997) for the study lake were based on temperatures reconstructed for nearby lake Nižné Terianske pleso (Battarbee et al. 1998). Reconstructed data were corrected for the elevation difference between lakes, applying an average annual lapse rate of − 5.2 °C/km, calculated from monthly upper-air temperature lapse rates of the atmospheric layer between 850 and 700 hPa (Agustí-Panareda and Thompson 2002). Recent air temperatures were recorded at the Skalnaté pleso meteorological station, and the same lapse rate was used for their correction. The overlap in recent and historical temperatures (1961–1997) enabled us to calibrate a model for the prediction of adjusted temperatures for the years 1998–2013. A simple linear model performed reasonably well (R^2^ = 0.86, F_(1,35)_ = 217, *p* < 0.0001) and showed good predictive accuracy (fivefold cross-validated root mean square error = 0.2 °C). This scaling allowed the recent temperature data to seamlessly continue the historical data. The final step for preparation of a climatic predictor variable was to smooth the temperature time series by means of locally weighted regression smoothing (LOESS) with a span of 0.5 (Cleveland et al. 1993). The smoothed values for the corresponding year of the midpoint of each sample were then used as a predictor in data analysis (hereafter temperature) (Fig. S2).

### Diatom Analysis

A total of 20 samples were analyzed for diatoms, evenly spaced down the core. The samples were processed according to standard methods (Battarbee 1986) using 30% H_2_O_2_, HCl, and distilled water. Diatom samples were mounted on slides in Naphrax with refractive index 1.710. Diatoms were identified under a light microscope with total magnification of × 1000, using oil immersion objectives. Approximately 400 diatom valves were counted on each slide. Identification and taxonomy were based on Krammer and Lange-Bertalot (1986, 1988, 1991a, b), Lange-Bertalot and Krammer (1989), and Coste and Rosebery (2011). Life forms of diatoms were assigned according to Denys (1991/2).

### Diatom-Inferred TP Reconstructions

Quantitative reconstruction of the diatom-inferred total phosphorus (DI-TP) was performed using the available European Diatom Database (EDDI) (estimates of TP, Juggins 2003, current data at http://craticula.ncl.ac.uk/Eddi/jsp/index.jsp). The modern analogue technique (MAT) was used to measure the similarity between each diatom sample and the training set, comparing them numerically using the squared chi-squared distance as the dissimilarity measure. The fossil samples outside a minDC value (i.e., the distance to the closest modern analogue for each fossil sample) of 100–150 have no good analogue in the training set. DI-TP was calculated using the combined TP dataset (derived from nine datasets, together 347 samples) (Juggins 2001), covering a TP range of 2–1189 μg L^−1^, mean 98.6 μg L^−1^. The weighted-averaging (WA) method was used, which has good empirical predictive ability (Juggins 2001).

### Data Analysis

The diatom species matrix of relative abundances was submitted to stratigraphically constrained incremental sum of squares cluster analysis (CONISS; Grimm 1987) in order to identify stratigraphic zones across which the diatom flora changed markedly. The number of significant zones was determined by the broken-stick model (Bennett 1996). Species characteristic for each zone were identified among the dominant taxa (> 5% in at least one of the samples) using indicator species analysis (Dufrêne and Legendre 1997). The indicator value (IndVal) of each species was tested using a randomization test with 10,000 permutations.

Redundancy analysis (RDA) was used to relate the composition of diatom assemblages to changes in climate and trophy of the lake. Temperature and diatom-inferred total phosphorus were used as predictors in RDA. However, in order to prevent circularity, diatom-inferred TP was used as a passive variable and was projected into the ordination in the directions of its maximal correlation with the configuration of sample scores. Thus, to not affect the results of the ordination analysis, DI-TP was added afterwards so that the trophic changes could still be judged from the ordination plot. Similarly, undated samples (pre-1814) were treated as passive in the analysis. Since the sediment samples form a time series, we investigated the variogram of the residual variance from the RDA model (Wagner 2004) and found a significant temporal autocorrelation. Thus, we accounted for the effect of serial correlation using a permutation test restricted for the time series.

Generalized linear models with the logit link-function and binomial error distribution (GLM, McCullagh and Nelder 1989) were used to test the effects of temperature, trophy (DI-TP), and their interactions on the proportion of life forms in diatom assemblages. Indeed, both DI-TP and proportions of life forms are derived from the same species matrix. However, all life form groups comprise species with a wide variety of TP optima and thus, we consider those quantities as independent in the analysis. Since the GLMs showed considerable overdispersion, standard errors were computed by a quasi-likelihood procedure. Again, the residuals were checked for independence, but no significant temporal autocorrelation was detected.

Finally, the effect of temperature on DI-TP and LOI was assessed using linear models. Since ordinary least square models showed significant autocorrelation at short time lags, linear models were re-fitted using generalized least squares (GLS) with a first order continuous autoregressive correlation structure (Pinheiro and Bates 2000).

All analyses were performed in R (R Core Team 2017) using the libraries indicspecies (De Caceres and Legendre 2009), nlme (Pinheiro et al. 2017), rioja (Juggins 2017), and vegan (Oksanen et al. 2017).

## Results

### Subfossil Diatom Assemblages

**Fig. 2:**
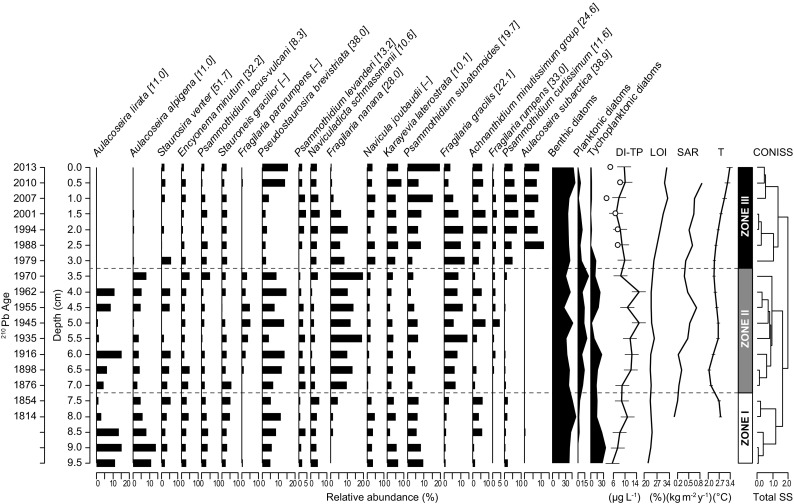
Stratigraphic changes in relative abundances of dominant diatom taxa (representing > 5% of abundance at least in one sample), relative abundances of diatom life forms, diatom-inferred total phosphorus (DI-TP), amount of organic matter (LOI), sediment-accumulation rate (SAR), and temperature (T) record/estimated for Popradské pleso lake. Error bars represent estimates of DI-TP and T ± 1 × standard error. Dashed lines delineate significant stratigraphic zones in the diatom assemblage as determined by constrained cluster analysis (CONISS). TP optima of the species are displayed in square brackets. Circles on the DI-TP graph represent measured TP values

A total of 122 diatom taxa representing 40 genera were identified, predominantly pennate species. Overall, the most abundant species were *Psammothidium subatomoides*, *Fragilaria nanana*, *Aulacoseira lirata*, and *Pseudostaurosira brevistriata*, together accounting for ~ 25% of the total abundance. Even though not as abundant, *Staurosira venter*, *Encyonema minutum*, *Psammothidium lacus-vulcani*, and *Stauroneis gracilior* were stable components of the assemblages over the whole sediment core. The proportion of 19 species was higher than 5% in at least one of the samples. Constrained cluster analysis identified three significant stratigraphic zones (Fig. 2).

Zone I (10 to 7.5 cm, ~ 1814 to 1854) represents the bottom part of the core and was characterized by the dominance of the centric diatoms *Aulacoseira alpigena* (IndVal = 67%, *p* = 0.0017) and *A*. *lirata* (IndVal = 59%, *p* = 0.0258). The proportions of *Pseudostaurosira brevistriata* and *Psammothidium subatomoides* were also high (~ 10%). There was also a subtle increase in the proportion of *Achnanthidium minutissimum* group to ca. 6%. Species such as *Stauroneis gracilior*, *Encyonema minutum*, small benthic species such as *Psammothidium lacus-vulcani* and *Staurosira venter*, and the small-celled *Naviculadicta schmassmanii* and *Navicula joubaudii* became relatively stable elements of the diatom assemblages (fluctuating around ~ 3%).

Zone II (7.5 to 3.0 cm, 1854 to 1979) was characterized by the occurrence of *Fragilaria pararumpens* (IndVal = 82%, *p* = 0.0003) and also by markedly high abundances (up to ~ 20%) of planktonic species, such as *Fragilaria nanana* (IndVal = 68%, *p* = 0.0001) and *F*. *gracilis* (IndVal = 48%, *p* = 0.0402). These species dominated together with the benthic *Pseudostaurosira brevistriata*, the relative abundance of which oscillated between 5 and 13%. The tychoplanktonic *Aulacoseira lirata* and *A*. *alpigena* still represented a numerically important part of the community, although both species decreased dramatically at the end of the zone. *Stauroneis gracilior*, *Encyonema minutum*, taxa including members of *Psammothidium*, *Karayevia*, *Navicula*, and *Naviculadicta* become notably less abundant.

Zone III (3.0 to 0 cm, 1979 to 2013) was characterized by elevated abundances of *Aulacoseira subarctica* (IndVal = 84%, *p* = 0.0005), *Psammothidium curtissimum* (IndVal = 76%, *p* = 0.0002), and *Karayevia laterostrata* (IndVal = 45%, *p* = 0.0106), which was accompanied by the disappearance of *Aulacoseira lirata* and a marked decrease of *A*. *alpigena* and *Fragilaria* species (*Fragilaria gracilis*, *F*. *nanana*, *F*. *rumpens*, and *F*. *pararumpens*). *Psammothidium subatomoides* became a major element of the assemblages with a relative abundance of ~ 15%.

### Diatom-Inferred TP Reconstructions

The combined TP diatom dataset fit best with the sediment core samples. The calculated values of the DI-TP were towards the oligotrophic end of the training set, therefore WA with classical deshrinking was applied. In general, a good agreement between modern and fossil diatom assemblages was found. The MAT analysis showed that the minDC values varied between 100 and 150. Three samples were outside the minDC range, indicating no good analogues in the modern training set (0.25–93 minDC, 0.75–86 minDC, 5.25–99 minDC). The DI-TP over the whole sediment core varied between 6 and 15 μg L^−1^ (Fig. 2). The average DI-TP values were lowest in Zone I (mean ~ 8 μg L^−1^, range ~ 6–10 μg L^−1^), while the highest values of DI-TP (mean ~ 12, range ~ 8–15 μg L^−1^) were calculated for Zone II. The average DI-TP of Zone III was ~ 8.8 μg L^−1^, range ~ 7–10 μg L^−1^.

Overall, the EDDI transfer function showed the following summary statistics for the TP model: r^2^ (apparent coefficient of determination of the regression of the predicted on the observed value) = 0.71, RMSE (apparent root mean square error of prediction) = 0.35, r^2^_jack-knife_ = 0.63, RMSE _jack-knife_ = 0.38, Ave Bias _jack-knife_ (average of residual) = 0.003, and Max Bias _jack-knife_ = 0.49.

### Environmental Drivers of Assemblage Composition

**Fig. 3:**
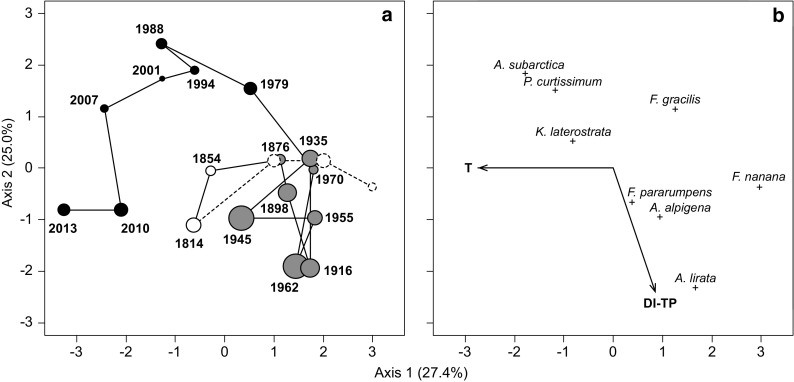
RDA ordination plot showing the time trajectory of diatom assemblages in Popradské pleso lake (**a**) and relationships between temperature (T), diatom-inferred total phosphorus (DI-TP) and species characteristic for the three significant stratigraphic zones (**b**). Three passive undated samples were projected into the RDA ordination plot (dashed circles). The size of the sample centroids is proportional to DI-TP values and the color of centroids indicates stratigraphic zones I (white circles), II (gray circles), and III (black circles). The percentage of variance explained by each axis is given in parentheses. Ordination scores are scaled symmetrically

Redundancy analysis showed a significant effect of temperature on the composition of diatom assemblages (pseudo-F = 5.66, *p* = 0.0294), and 27.4% of the variation in the species matrix can be accounted for by temperature. The effect of temperature was manifested mainly in Zone III where the assemblage trajectory considerably shifted along the first ordination axis (Fig. 3). On the other hand, patterns along the diagonal of the ordination space may be largely attributed to the trophic status of the lake. The stratigraphic zonation clearly tracks the variation in DI-TP.

**Fig. 4:**
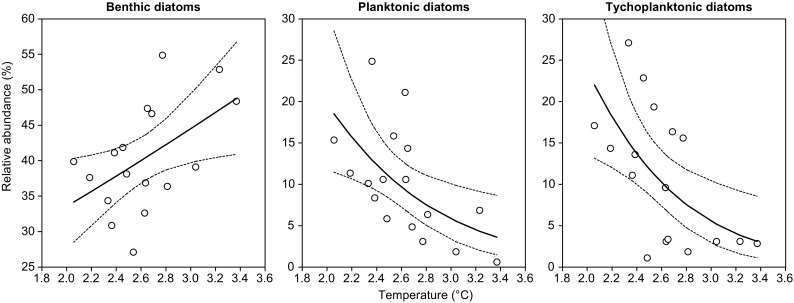
Relationships between relative abundance of diatom life forms and temperature in Popradské pleso. Significant GLMs (solid lines) along with 95% confidence bands (dashed lines) are displayed

The considerable effect of climate was also evident in the proportions of diatom life forms (Fig. 2). The relative abundance of benthic species significantly increased with increasing temperatures (GLM, F_1,15_ = 5.31, *p* = 0.036), while proportions of planktonic (GLM, F_1,15_ = 7.59, *p* = 0.0147) and tychoplanktonic species (GLM, F_1,15_ = 8.75, *p* = 0.0098) showed opposite patterns (Fig. 4). This effect of temperature was independent of changes in lake trophy (non-significant temperature × DI-TP interactions), and the effect of DI-TP was non-significant.

After accounting for a serial correlation, we did not find any statistical effect of temperature on DI-TP (GLS, F_1,15_ = 0.12, *p* = 0.7366) and LOI (GLS, F_1,15_ = 1.85, *p* = 0.1939).

## Discussion

During the whole studied period, the diatom assemblages of Popradské pleso were composed of a relatively balanced mixture of planktonic and periphytic communities. However, benthic communities slightly dominated, most likely because of the extent of the littoral zone and permanently high water transparency.

The oldest recorded diatom assemblages (until the mid-nineteenth century) dominated by *Aulacoseira lirata* and *A*. *alpigena* can be considered as a remnant of the diatom community surviving under cooler climatic conditions during and shortly after the Little Ice Age (LIA) that finished in the Tatra Mts. around 1895 (Kedzia & Kotarba 2018). Both heavily silicified tychoplanktonic species require turbulent water column to remain in the photic zone (Rühland et al. 2015) and they are indicative of windier conditions and increased water mixing as well as shorter and weaker stratification (Lotter et al. 2010). Moreover, a longer duration of ice cover trapping CO_2_ within the lake could have led to a decrease in lake water pH supporting *Aulacoseira* species that are more common in slightly acidic waters (Rühland and Smol 2005).

The shift to *Fragilaria* dominated assemblage was gradual and *Fragilaria nanana* and *F*. *capucina* sensu lato (including *F*. *gracilis*, *F*. *rumpens*, and *F*. *pararumpens*) reached maxima in their relative abundance between the mid-1920s and the end of the 1970s. This is consistent with the findings of Juriš et al. (1965) who recorded the dominance of *F*. *nanana* in the phytoplankton community in 1961–1963. This change in diatom assemblages can be attributed to increased temperatures after the LIA, although the effect of temperature seems to be weak within the first and second zone (Fig. 3). However, in alpine lakes, even a slight increase in temperature could induce the recorded changes in diatom assemblages (Smol et al. 2005). Numerous studies documented an increase in pennate planktonic *Fragilaria* in response to modern warming (Michelutti et al. 2015).

Besides temperature, nutrient concentration may play an important role in driving diatom communities (Lotter et al. 1998; Bennion et al. 2012). The predominance of *Fragilaria* species in the second zone could have been a response to the high density of trout in the lake. *Fragilaria* prefers more fertile conditions and may have benefited from the phosphorus released by fish while its amount increased proportional to fish biomass (Griffiths 2006). The native trout population in Popradské pleso was undoubtedly repeatedly manipulated in an uncontrolled manner to make the site more attractive for visitors, as angling was not prohibited until the declaration of the Tatra National Park in 1949. The only paper giving specifics on the fish stock refers to an introduction of 5000 brown trout and 1000 non-indigenous rainbow trout to the lake in 1934 (Dyk 1960). During his investigation in 1950s, Dyk (1960) considered the fish stock to be over-populated, and this situation likely lasted until the early 1960s or even longer (Zontág and Kot 2010). A similar situation was recorded in the lake outlets of the Polish Tatra Mts., when the high abundance of benthic *Fragilaria capucina gracilis* group was related to increased nutrient loads from non-native trout populations (Kawecka and Robinson 2008) and supports our interpretation.

The shift towards the recent diatom assemblage dominated by tychoplanktonic *Aulacoseira subarctica* and some benthic taxa (*Psammothidium curtissimum*, *P*. *subatomoides*, *Karayevia laterostrata*) was the most remarkable change in the community structure, especially considering the straightforward movement along the temperature gradient towards warm conditions (Fig. 3). The assemblage has begun to form since the beginning of the 1960s simultaneously with subfossil cladocerans and chironomids (Dobríková et al. 2016); however, compared to cladocerans that reacted instantly, the diatoms changed gradually until the mid-1970s, when their community composition stabilized. Although the timing of the shift corresponds with the beginning of the direct pollution of the lake by wastewater from the hotel (Juriš et al. 1965), the compositional change is not consistent either with the expected direct sewage influx or the transfer of the wastewater pipeline close to the outlet in 1994. Instead, our results suggest that rather climate-related factors were driving the diatom communities of the lake, and trophic-related variables played secondary role in the community changes.

In the Tatra Mts., the trend and amplitude of warming have been similar to that in other European regions (Berthon et al. 2014), and the annual mean temperature has increased by 1.1 °C during the last 20 years (based on our unpubl. data, Fig. S2).

An increase in water temperature resulted in longer ice-free periods and associated limnological changes, such as longer duration and extent of thermal stratification, and increased nutrient cycling (Rühland & Smol 2005). Under such conditions, *Aulacoseira lirata* and *A*. *alpigena* disappeared and *Fragilaria* species progressively declined, while *Aulacoseira subarctica* expanded during the last decades. *A*. *subarctica* is able to survive under low-light conditions and benefits from short, mild winters, and a long-lasting spring circulation (Horn et al. 2011). It flourishes at intermediate phosphorus concentrations (Gibson et al. 2003), and in some cases, its increase suggests biological recovery from eutrophication (Bennion et al. 2012).

Further evidence for recent warming is suggested by an increase in percent LOI that is consistent with the highest increase of sediment accumulation rate observed in more recent periods in European lakes, caused by significant anthropogenic influences and additional climate warming (Rose et al. 2011).

The response of the most recent diatom assemblage of Popradské pleso to warming contradicts the results of most paleolimnological studies carried out in the Northern Hemisphere. Common to all of these studies is an unprecedented shift in the diatom records from the benthic to planktonic life strategy linked to an increase in temperature and the strengthening of thermal stratification (Smol et al. 2005). In case that the diatom community is dominated by benthic forms, changes induced by warmer climate are subtler (Rühland and Smol 2005). Surprisingly, our study shows the prevalence of benthic diatoms throughout the full analyzed history of the lake and even a significant positive relationship between relative abundance of benthic species and increasing temperature.

As planktonic diatoms require stratification of the water column to maintain their position in the photic zone, their absence or low abundance suggests that a lake may not thermally stratify, or may only become weakly stratified during the open water season, or it is too shallow to support large planktonic diatom communities (Karst-Riddoch et al. 2009). Therefore, we suppose that the low representation of planktonic species in the diatom assemblages could be a result of weak stratification as a consequence of water currents induced by the strong inflow stream into the lake. It has been shown that the inflow stream had significant effect on the lake biota of Popradské pleso in the past (Hamerlík et al. 2016).

Diatom-inferred TP reconstructions have been firstly adopted in the Tatra Mountain lakes. We found that the variation in DI-TP followed closely the stratigraphic zonation. Contrary to general expectation, the diatom-inferred TP indicated more fertile lake conditions already in the second half of the nineteenth century, pre-dating the supposed impact of the sewage discharge. The change in the reconstructed TP values appeared when *Fragilaria* species gained more importance in the stratigraphic record. As mentioned above, the start of the shift in diatom assemblages could be related to climate warming. However, the diatom population increase was supported by phosphorus input released from over-populated trout population combined later with sewage influx. The highest values of DI-TP were recorded in the 1940s and 1960s, indicating mesotrophic conditions according to the OECD (1982) standards (DI-TP above 14 μg L^−1^).

The DI-TP values indicate re-oligotrophication since the end of the 1960s and the beginning of the 1970s despite the mitigative measures in 1994 (i.e., the transfer of the wastewater pipeline close to the outlet).

Unfortunately, more intense limnological studies of Popradské pleso started only after eutrophication had been recognized as an environmental issue, i.e., after 1960, and thus, due to the lack of instrumental measurements of TP, it is not possible to confirm the reliability of the TP reconstruction prior to that period. The concentrations of orthophosphate P in Popradské pleso varied from 2 to 28 μg L^−1^ in 1961–1963 (Vranovský 1991), and TP ranged from 1.5 to 16 μg L^−1^ in 1980–2016 (Kopáček, unpubl. data). This latter data are from a single measurement per year carried out in autumn. As TP can be highly variable both intra- and inter-annually (e.g., Bennion et al. 2000), comparing these values with the model that provides an estimate of mean TP concentrations usually encompassing several years may be rather misleading. Despite it, our DI-TP values are generally in agreement with the instrumental records, although they are somewhat higher as DI-TP models often overestimate phosphorus in less productive environments (Sayer 2001 and citations therein).

## Conclusions

1. Major changes in the diatom assemblages were not in line with our hypothesis. The timing of the first significant shift in the assemblages in the second half of the nineteenth century precludes the possibility of being caused by direct human activities and was most likely the effect of post-LIA warming and amplified by fish manipulations.
2. The diatom data suggest that the most significant change in the lake ecosystem was mainly related to climate warming that masked the seemingly straightforward effect of waste waters, especially after the 1970s.
3. The positive relationship between the relative abundance of benthic species and increasing temperature is contradictory with other studies and could be a result of weak stratification of the water column as a consequence of strong currents induced by the inflow stream.

## Electronic supplementary material

Fig. S1 Depth-age model of the 0–8 cm section of the sediment based on ^210^Pb and ^137^Cs dating from (Hamerlík et al. 2016) (PNG 13 kb)

High resolution image (TIF 689 kb)

Fig. S2 Mean annual air temperatures at Popradské pleso reconstructed for 1814–2013 and adjusted after Agustí-Panareda & Thompson (2002). Overall temperature trend is depicted by the thick LOESS curve with a span of 0.5 (± standard error, dashed lines) (PNG 114 kb)

High resolution image (EPS 588 kb)
