# Supplementary figures and images for: Reconstructing the Trophic History of an Alpine Lake (High Tatra Mts.) Using Subfossil Diatoms: Disentangling the Effects of Climate and Human Influence
Source: Water Air Soil Pollut. 2018 Aug 15;229(9):289. doi: 10.1007/s11270-018-3940-9 (PMC6096542; doi:10.1007/s11270-018-3940-9)

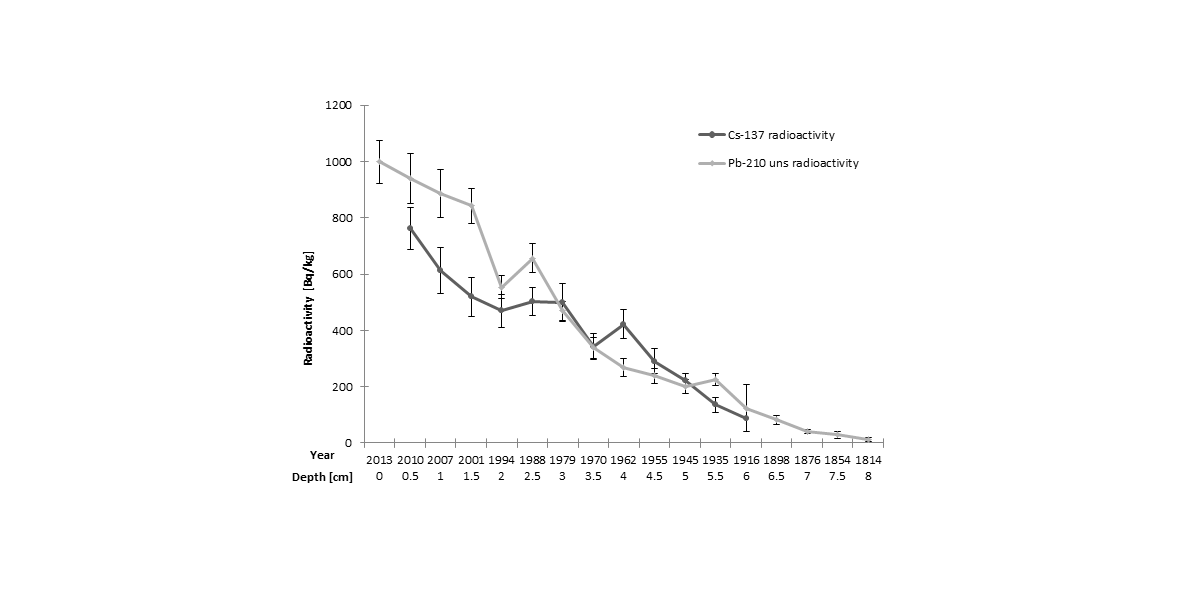

Supplement: Supplementary file 1 — Depth-age model of the 0–8 cm section of the sediment based on 210Pb and 137Cs dating from (Hamerlík et al. 2016) (PNG 13 kb) [file 11270_2018_3940_Fig5_ESM.png]

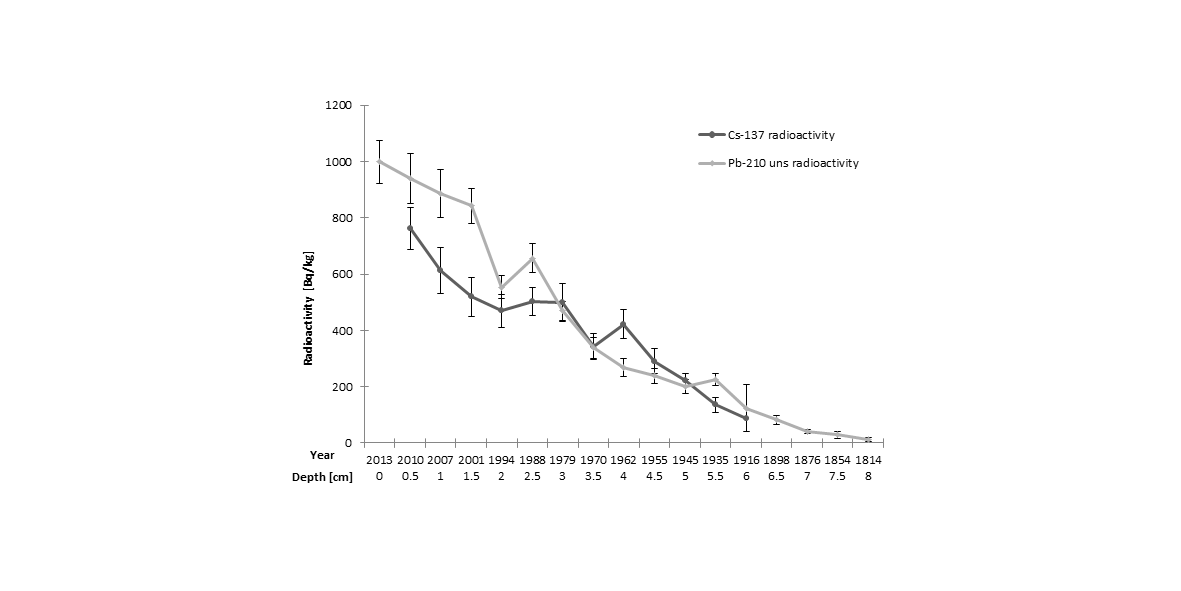

Supplement: Supplementary file 2 — High resolution image (TIF 689 kb) [file 11270_2018_3940_MOESM1_ESM.tif]

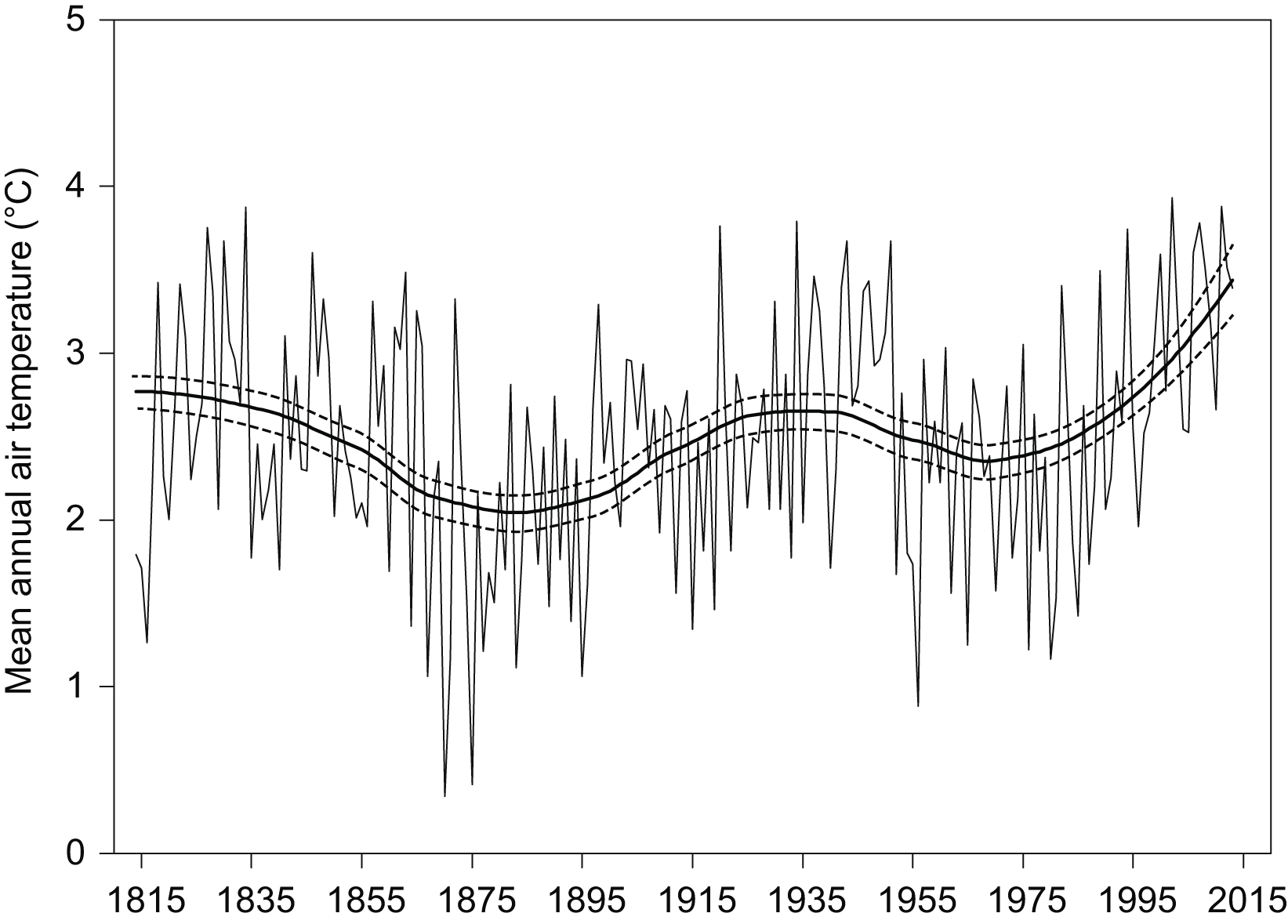

Supplement: Supplementary file 3 — Mean annual air temperatures at Popradské pleso reconstructed for 1814–2013 and adjusted after Agustí-Panareda & Thompson (2002). Overall temperature trend is depicted by the thick LOESS curve with a span of 0.5 (± standard error, dashed lines) (PNG 114 kb) [file 11270_2018_3940_Fig6_ESM.png]
